# A novel treatment for radiation proctopathy using monopolar spray coagulation with a polypectomy snare tip

**DOI:** 10.1055/a-2598-4404

**Published:** 2025-05-28

**Authors:** Diego Cadena-Aguirre, Luciano Lenz, Marcelo Simas de Lima, Amanda Aquino Miranda Pombo, Rafael Sartori Balbinot, Adriana Vaz Safatle-Ribeiro, Fauze Maluf-Filho

**Affiliations:** 1 215027 Instituto do Câncer do Estado de São Paulo, University of São Paulo, São Paulo, Brazil

Radiation proctopathy (RP) is recognized as injury to the rectum due to pelvic radiotherapy 1 . A variety of endoscopic therapies have been used for the management of bleeding from chronic RP, and argon plasma coagulation (APC) is probably the most widely used technique 2 . Spray coagulation (SC) is a novel, non-contact technique for treating vascular lesions. Like APC, SC uses monopolar diathermy to achieve precise, superficial tissue penetration through the tip of a conventional polypectomy snare 3 . However, SC offers several advantages, such as eliminating the need for argon gas, specialized catheters, and dedicated generators, making it more accessible and easier to perform 1 .

During APC, bowel distension and catheter malfunction can occur. The snare-tip SC technique addresses these challenges by providing comparable thermal effects using only a polypectomy snare and spray-mode coagulation (Effect 1, 40 watts). Preclinical data suggest that SC achieves tissue penetration like APC, ensuring effective hemostasis while maintaining a high level of safety profile 4 .

This technique reduces equipment dependency and procedural costs, making it an attractive option, especially in resource-limited settings. The snare-tip SC technique requires a 2–3 mm distance from the lesion. Two configurations are possible: exposing 1–2 mm of the snare tip, so the beam spreads laterally ( Fig. 1 **a, c** ), or keeping the snare tip inside the sheath, so the beam spreads forward ( Fig. 1 **b, d** ) 5 .

**Fig. 1:**
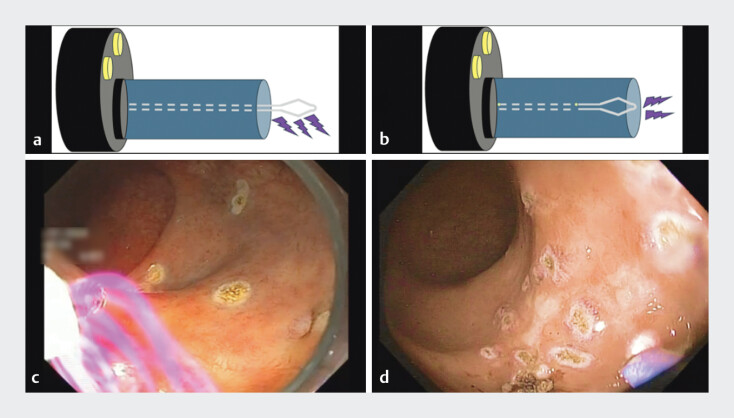
Different beam spreads according to snare-tip exposure. **a, c** Minimal snare-tip exposure (1–2 mm) with lateral beam spreading. **b, d** Snare tip under the sheath with forward beam spreading.

The next video ( Video 1 ) shows a male patient with rectal bleeding after radiotherapy ( Fig. 2 ) due to prostate cancer and aims to show the snare-tip SC technique for treating RP ( Fig. 3 ). Follow-up colonoscopy after 4 weeks shows healing ulcers with adequate endoscopic and clinical response ( Fig. 4 ). More clinical studies are needed to establish its safety and efficacy.

Treatment for radiation proctopathy using monopolar spray coagulation with a polypectomy snare tip.Video 1

**Fig. 2:**
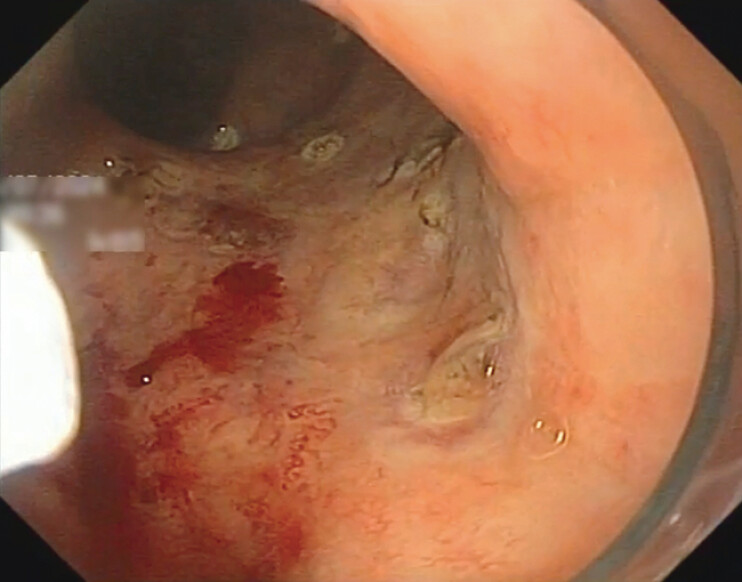
Oozing bleeding due to radiation proctopathy.

**Fig. 3:**
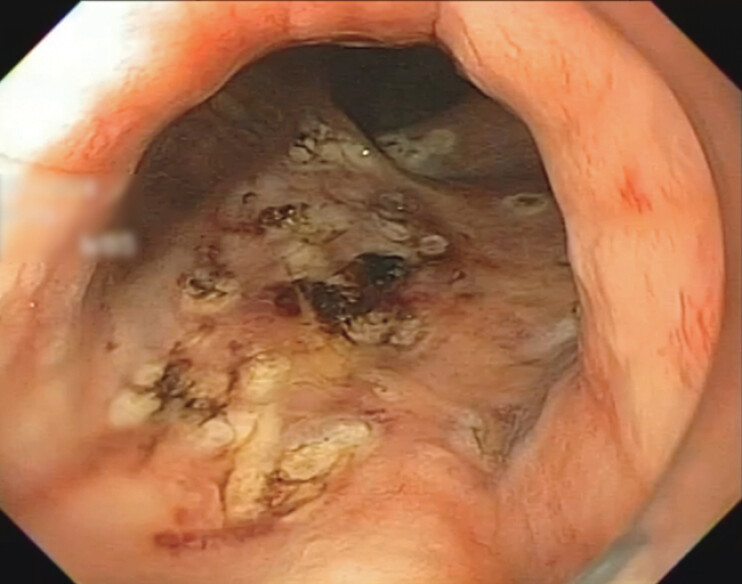
Final aspect after snare-tip spray coagulation of rectal angioectasia.

**Fig. 4:**
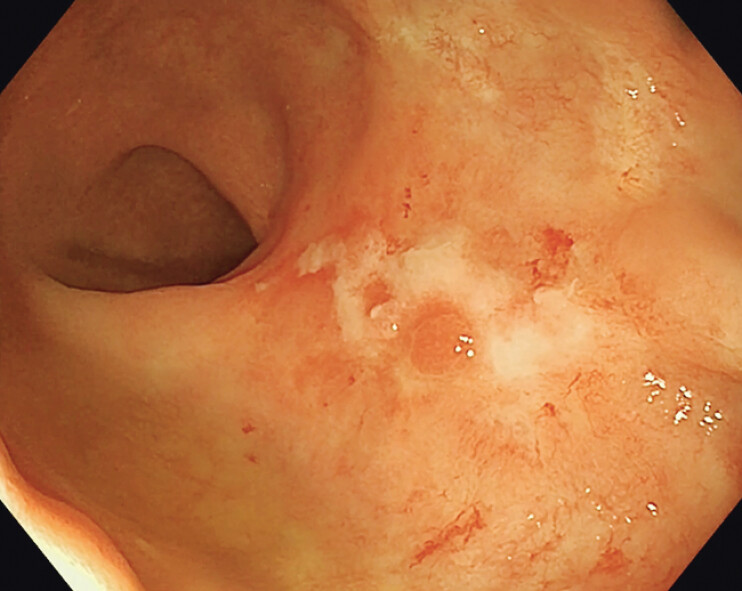
Follow-up colonoscopy after 4 weeks of spray coagulation hemostasis.

Endoscopy_UCTN_Code_TTT_1AQ_2AZ

## Notes

Endoscopy E-Videos https://eref.thieme.de/e-videos
*E-Videos* is an open access online section of the journal *Endoscopy* , reporting on interesting cases and new techniques in gastroenterological endoscopy. All papers include a high-quality video and are published with a Creative Commons CC-BY license. Endoscopy E-Videos qualify for HINARI discounts and waivers and eligibility is automatically checked during the submission process. We grant 100% waivers to articles whose corresponding authors are based in Group A countries and 50% waivers to those who are based in Group B countries as classified by Research4Life (see: https://www.research4life.org/access/eligibility/ ). This section has its own submission website at https://mc.manuscriptcentral.com/e-videos .
